# Determinants of Smoking Among University Students in Northern Iraq

**DOI:** 10.34172/jrhs.9009

**Published:** 2025-06-10

**Authors:** Mehdi Mirzaei-Alavijeh, Rebwar Rzgar Qadir, Negar Karimi, Farzad Jalilian

**Affiliations:** ^1^Social Development and Health Promotion Research Center, Health Policy and Promotion Research Institute, Kermanshah University of Medical Sciences, Kermanshah, Iran; ^2^Department of Health Education and Promotion, School of Health, Kermanshah University of Medical Sciences, Kermanshah, Iran; ^3^Family Health and Population Growth Research Center, Health Policy and Promotion Research Institute, Kermanshah University of Medical Sciences, Kermanshah, Iran

**Keywords:** Smoking, Students, Peer influence, Iraq

## Abstract

**Background:** Smoking remains a serious public health issue on a global scale and warrants increased attention. This research aimed to assess the prevalence of smoking and identify key predictors driving the adoption of smoking prevention behaviors among university students in northern Iraq.

**Study Design:** A cross-sectional study.

**Methods:** An online study was conducted among 765 students at Raparin University, Sulaymaniyah, Iraq. The required data were collected using a structured questionnaire distributed through Google Forms. The questionnaire, developed from standardized instruments, assessed sociodemographic factors and determinants of smoking behaviors. Finally, the data were analyzed by SPSS-16 using linear and logistic regressions.

**Results:** The average age of the students was 21.04 years [95% confidence interval: 20.89, 21.20], with ages ranging from 17 to 29 years. The age group of 21–23 years old increased the chances of cigarette smoking among students (odds ratio [OR]: 2.068). In addition, male students were more likely to have cigarette smoking (OR: 11.675). Father smoking, brother smoking, and friend smoking increased the chances of cigarette smoking by 1.981, 2.687, and 10.426 times among students, respectively. Our study identified key determinants of smoking preventive behaviors, including peer pressure (B=0.507), self-image (B=0.235), belief (B=0.134), value (B=0.184), attitude (B=0.115), and the influence of friends who smoke (B=-1.110).

**Conclusion:** Our findings emphasize the critical roles of peer pressure and self-image in influencing smoking behaviors among students. To address this issue, targeted educational programs that foster positive self-image and resilience against peer influence are vital for effective smoking prevention strategies.

## Background

 According to the report by the World Health Organization, the number of tobacco users was 1.32 billion in 2015, dropped to 1.30 billion in 2020, and is projected to further decline to around 1.27 billion by 2025. While this downward trend is encouraging, it does not overshadow the staggering reality that tobacco use still claims 8.7 million lives annually.^1^ This firmly positions smoking as the leading cause of preventable illness and death.^2^ In developing countries, however, smoking remains an overlooked health issue and a significant driver of adult mortality and morbidity, with 80% of tobacco-related deaths and health complications concentrated in these regions.^3,4^ Tobacco use is among the most critical health challenges of our era, responsible for nearly 98% of lung cancer deaths and over 80% of chronic obstructive pulmonary disease cases.^5^ Tobacco use is a modifiable behavioral risk factor linked to numerous diseases and disabilities; smoking harms every organ in the body and contributes to several life-threatening conditions, including cancer, cardiovascular diseases, and chronic obstructive pulmonary diseases.^6^ Research indicates that smoking reduces life expectancy by an average of seven years.^7^ Additionally, evidence points to an increased risk of psychiatric disorders, such as suicide and bipolar disorder, among smokers.^8,9^ Both tobacco use and major depression significantly contribute to the global disease burden and often occur together.^9^ Beyond its health impacts, smoking negatively affects academic performance among students.^10,11^ Furthermore, the harmful effects of smoking are not confined to smokers alone, as nonsmokers are also at risk through exposure to second-hand smoke.^12^

 Smoking remains a critical public health issue on a global scale and requires increased attention.^13^ To curb its prevalence among students, it is essential to examine how their attitudes toward smoking develop and identify factors that shape their smoking behaviors.^14^ Gaining deeper insights into factors driving smoking initiation and cessation can support the creation of more effective strategies to facilitate changes in smoking behavior.^15^ Understanding what influences individuals to start smoking may help deter vulnerable populations from ever picking up their first cigarette. ^6^ There are numerous theories focused on understanding behaviors, attitudes, and changes in behavior, which can be effectively applied in health promotion programs; using theory-based interventions offers several benefits, such as pinpointing essential constructs to address and choosing suitable techniques for intervention.^16^ This study was conducted within the framework of the Integrative Model of Factors Influencing Smoking Behaviors developed by Flay et al. The model explores how various determinants, such as knowledge, attitudes, beliefs, values, intentions, family and peer influences, and self-image, affect smoking behavior.^17^ Considering the scarce evidence regarding factors influencing the adoption of smoking prevention behaviors in Iraq, this research seeks to assess the prevalence of smoking and identify key predictors driving the adoption of smoking prevention behaviors among university students in northern Iraq.

## Methods

###  Study design 

 An online cross-sectional study was performed among 765 students at Raparin University, located in Sulaymaniyah in northern Iraq. The research took place between December 2022 and January 2023. The participants were limited to Raparin University students, as defined by the inclusion criteria. The required sample size was calculated drawing from a previous study by Qudah in Iraq,^18^ which reported a smoking prevalence of 19.4% among the students. Using a 95% confidence level (CI) and an accuracy margin of 0.03, the estimated sample size was 695 participants. To account for a potential 10% rejection rate, a total of 765 students were examined in this study.

###  Questionnaire 

 Google Forms were utilized to gather data, sharing an invitation link with a survey questionnaire via email to the university students through their respective teachers. The questionnaire was developed based on standardized questionnaires.^19-21^ To ensure the authenticity of responses and prevent duplication, students were instructed to complete the survey only once. The participants were informed about the confidentiality of their responses, assured that participation was voluntary, and notified that the questionnaire had been specifically designed by the authors for this study.

 The questionnaire was structured into two sections. The first section consisted of 15 questions aimed at collecting sociodemographic data, including variables such as gender, age, academic year, marital status, parental education, family economic status, living arrangements, and smoking habits of the students. The second section comprised 33 questions designed to evaluate various determinants of the Integrative Model of Factors Influencing Smoking Behaviors.

 This section included six items assessing knowledge about the health effects of smoking (e.g., awareness that smoking causes lung cancer). Seven items focused on attitudes toward smoking prevention behaviors (e.g., supporting smoking bans in public spaces such as restaurants). Beliefs about smoking prevention behaviors were measured with three items (e.g., convictions about health risks such as cancer from smoking). Values related to smoking prevention behaviors were assessed with three items (e.g., acknowledging that smoking harms the body). Intentions toward smoking prevention behaviors were captured using two items (e.g., contemplating starting to smoke within the next two months). Family influence (e.g., likelihood of accepting a cigarette offered by a family member) and peer influence (e.g., accepting a cigarette offered by a close friend) were evaluated using two items for each. Additionally, self-image related to smoking was examined using four items (e.g., believing that smoking could lower one’s self-esteem). Finally, smoking prevention behaviors were measured through four items (e.g., leaving an area where someone is smoking nearby).

 To ensure the clarity and accuracy of the questionnaire, a research team conducted a thorough review focusing on its readability and content. A pilot test was subsequently performed to assess its reliability and validity. After making necessary adjustments, a certified English teacher translated the questionnaire into Kurdish to facilitate its use by the target population. The face validity of the questionnaire was evaluated using qualitative methods, which included individual face-to-face interviews with 12 experts from diverse fields, such as public health, psychology, health education, health promotion, and health policy. Feedback from these experts was analyzed, leading to further modifications of the questionnaire based on their insights. Both quantitative and qualitative approaches were utilized for content validity. Similar to the face validity assessment, another group of 12 experts provided feedback regarding the difficulty, relevance, and clarity of the questions. Their recommendations were taken into account, resulting in additional changes. Subsequently, these experts classified each question as essential, useful but not essential, or not essential. The collected feedback was then used to compute the content validity ratio (CVR) and content validity index (CVI). According to the Lawshe table, the minimum acceptable values for CVR and CVI were established at 0.62 and 0.79, respectively.^22,23^ The evaluation results confirmed that the questionnaire met the necessary standards for both CVR and CVI. Reliability was assessed using Cronbach’s alpha coefficient, with a value above 0.65 generally considered acceptable.^24^ Prior to the main study, a pilot study was conducted involving 30 participants from the target group. During this phase, Cronbach’s alpha coefficients were calculated, and the results indicated acceptable reliability for all determinants, including knowledge (α = 0.735), attitude (α = 0.803), belief (α = 0.798), value (α = 0.610), intention (α = 0.899), family influence (α = 0.636), peer influence (α = 0.866), self-image (α = 0.748), and smoking preventive behaviors (α = 0.698).

###  Statistical analysis

 The obtained data were analyzed using SPSS, version 16. Descriptive statistics, including frequencies and percentages, were utilized. Categorical data were summarized, and means and standard deviations (SD) were calculated for continuous variables. Bivariate correlations were assessed to determine the strength and direction of relationships between variables within the Integrative Model of Factors Influencing Smoking Behavior. Internal consistency for the various measures was evaluated using Cronbach’s coefficient alpha. To explore factors associated with smoking among college students, univariate analysis was first conducted through logistic regression, with non-significant variables subsequently excluded from the model. The results of the multivariate analysis were also reported. Additionally, hierarchical multiple linear regression analysis was performed to examine variations in smoking preventive behaviors, incorporating variables from the Integrative Model along with background characteristics. Statistical significance was defined as a *P*-value of less than 0.05.

## Results

 The average age of the students was 21.04 years (95% CI: 20.89, 21.20), with an age range of 17–29 years. Table 1 provides a comprehensive overview of the demographic characteristics of the student population surveyed, revealing significant insights into their age, gender, academic level, and smoking status. The majority of participants were within the age group of 17–20 years (46%), followed closely by those aged 21–23 (42.7%). Gender representation showed a higher percentage of females (63.4%) compared to males (36.6%). In terms of socioeconomic factors, a significant majority of students (79.1%) did not work while studying. Family economic status was predominantly reported as good (81.4%). The educational levels of parents revealed notable disparities, particularly with mothers, where 46.9% were reported as illiterate. The smoking status of the students demonstrated that 11.1% currently smoked, with a notable percentage of friends (42.2%) being smokers. Moreover, the data showed that 26.1% of fathers, 28.5% of brothers, 1.2% of mothers, and 0.4% of sisters were smokers.

**Table 1:** Demographic characteristics of the students

**Variables**	**Number**	**Percent**
Age		
17-20	352	46.0
21-23	327	42.7
24-26	61	8.0
27-29	25	3.3
Gender		
Male	280	36.6
Female	485	63.4
Academic level		
First year	194	25.4
Second year	195	25.5
Third year	138	18.0
Fourth year	238	31.1
Working beside study		
Yes	160	20.9
No	605	79.1
Family economic status		
Very bad	6	0.8
Bad	29	3.8
Good	623	81.4
Very good	107	14.0
Father’s level of education		
Illiterate	144	18.8
Primary school	247	32.3
Secondary school	161	21.0
High school	94	12.3
University degree	119	15.6
Mother’s level of education		
Illiterate	359	46.9
Primary school	258	33.7
Secondary school	76	9.9
High school	39	5.1
University degree	33	4.3
Marital status		
Single	692	90.5
Married	73	9.5
Residency		
Home	470	61.4
Dormitory	295	38.6
Smoking status		
Yes	85	11.1
No	680	88.9
Father smoker		
Yes	200	26.1
No	565	73.9
Mother smoker		
Yes	9	1.2
No	756	98.8
Sister smoker		
Yes	3	0.4
No	762	99.6
Brother smoker		
Yes	218	28.5
No	547	71.5
Friend smoker		
Yes	323	42.2
No	442	57.8

Table 2 presents a comprehensive analysis of factors influencing cigarette smoking among participants, highlighting both crude and adjusted odds ratios (ORs) for various demographic and social variables. Notably, gender emerged as the most significant factor, with males having an adjusted OR of 11.675 (4.769–28.579), indicating that men were approximately 11.7 times more likely to smoke compared to women (*P* < 0.001). In addition, the presence of smoking friends significantly influenced smoking behavior, with an adjusted OR of 10.426 (3.547–30.647), suggesting that having friends who smoke increased the likelihood of an individual smoking by over 10 times (*P* < 0.001). The father’s smoking status also had a critical role, with an adjusted OR of 1.981 (1.109–3.538), representing that individuals with a smoking father were nearly twice as likely to smoke themselves (*P* = 0.021). The analysis confirmed that individuals aged 21–23 had a crude OR of 2.581 for smoking, which decreased to 2.068 after adjustment. Older age groups demonstrated diminishing associations, with the 24–26 and 27–29 age groups having an adjusted OR of 1.665 and an OR of 0.767, respectively, implying no significant relationship with smoking. The adjusted ORs were calculated while controlling for several variables, including age, gender, academic level, employment status, family economic status, parental education levels, marital status, living situation, and the smoking status of family members. This adjustment allows for a clearer understanding of the independent effects of these factors on smoking behavior, isolating the impact of each variable while accounting for potential confounding influences.

**Table 2:** Factors related to cigarette smoking among the participants

**Variables**	**Crude OR (95% CI)**	***P *** **value**	**Adjusted OR (95% CI)**	***P *** **value**
Age (years)				
17-20	1.000		1.000	
21-23	2.581 (1.136, 4.381)	0.001	2.068 (1.104, 3.872)	0.023
24-26	3.673 (1.363, 7.892)	0.001	1.665 (0.648, 4.275)	0.290
27-29	2.045 (0.568, 7.365)	0.274	0.767 (0.179, 3.292)	0.722
Gender				
Female	1.000		1.000	
Male	26.368 (11.959, 58.135)	0.001	11.675 (4.769, 28.579)	0.001
Academic level				
First year	1.000		1.000	
Second year	1.106 (0.583, 2.100)	0.757	-	-
Third year	1.222 (0.615, 2.429)	0.567	-	-
Fourth year	1.067 (0.567, 1.976)	0.837	-	-
Working beside study				
No	1.000		1.000	
Yes	3.698 (2.310, 5.919)	0.001	1.078 (0.578, 2.011)	0.813
Family economic status				
Very bad	1.000		1.000	
Bad	1.042 (0.099, 10.959)	0.973	-	-
Good	0.534 (0.062, 4.721)	0.580	-	-
Very good	1.011 (0.111, 9.181)	0.992	-	-
Father’s level of education				
Illiterate	1.000		1.000	
Primary school	0.643 (0.335, 1.234)	0.185	-	-
Secondary school	0.578 (0.274, 1.217)	0.149	-	-
High school	1.151 (0.546, 2.426)	0.711	-	-
University degree	1.096 (0.542, 2.218)	0.798	-	-
Mother’s level of education				
Illiterate	1.000		1.000	
Primary school	0.643 (0.234, 1.768)	0.393	-	-
Secondary school	0.682 (0.244, 1.908)	0.466	-	-
High school	1.050 (0.338, 3.263)	0.933	-	-
University degree	0.467 (0.103, 2.121)	0.324	-	-
Marital status				
Single	1.000		1.000	
Married	0.835 (0.370, 1.884)	0.664	-	-
Staying place				
Home	1.000		1.000	
Dormitory	1.648 (1.048, 2.592)	0.030	1.741 (0.997, 3.042)	0.051
Father smoker				
No	1.000		1.000	
Yes	2.185 (1.371, 3.482)	0.001	1.981 (1.109, 3.538)	0.021
Mother smoker				
No	1.000		1.000	
Yes	2.317 (0.473, 11.337)	0.300	-	-
Sister smoker				
No	1.000		1.000	
Yes	4.036 (0.362, 44.985)	0.257	-	-
Brother smoker				
No	1.000		1.000	
Yes	2.373 (1.499, 3.757)	0.001	2.687 (1.501, 4.810)	0.001
Friends smoker				
No	1.000		1.000	
Yes	36.651 (13.268, 101.243)	0.001	10.426 (3.547, 30.647)	0.001

*Note*. OR: Odds ratio; CI: Confidence interval.

Table 3 provides an integrative model of factors influencing smoking behavior, highlighting the correlations among various variables. Based on the mean scores, knowledge (X1) had a positive correlation with attitude (X2, r = 0.207), belief (X3, r = 0.300), and other factors, suggesting that increased knowledge may enhance positive attitudes and beliefs regarding smoking preventive behaviors. Notably, values (X4) showed strong correlations with belief (r = 0.746), family pressure (X5, r = 0.427), and peer pressure (X6, r = 0.441), underlining that personal values significantly influence social pressures and beliefs about smoking preventive behaviors. Furthermore, intention (X8) demonstrated significant positive correlations with attitude (r = 0.585), peer pressure (r = 0.584), and self-image (X7, r = 0.578), representing that a strong self-image and positive social influences can enhance individuals’ intentions to engage in smoking preventive behaviors. Overall, the significant correlations across these variables underscore the complexity of smoking preventive behaviors, where knowledge, attitudes, beliefs, social pressures, and self-image interact to influence intentions and preventive behaviors related to smoking preventive behaviors.

**Table 3:** Integrative model of factors influencing the correlation among smoking behavior variables

**Variables**	**Mean (SD)**	**X1**	**X2**	**X3**	**X4**	**X5**	**X6**	**X7**	**X8**
X1. Knowledge	5.47 (1.04)	1.000							
X2. Attitude	30.78 (4.68)	0.207	1.000						
X3. Belief	12.98 (2.32)	0.300	0.636	1.000					
X4. Value	12.94 (2.32)	0.341	0.567	0.746	1.000				
X5. Family pressure	9.25 (1.59)	0.214	0.559	0.481	0.427	1.000			
X6. Peer pressure	8.56 (2.08)	0.273	0.586	0.503	0.441	0.653	1.000		
X7. Self-image	15.87 (3.83)	0.193	0.638	0.523	0.484	0.440	0.527	1.000	
X8. Intention	8.85 (1.73)	0.166	0.585	0.432	0.384	0.530	0.584	0.578	1.000
X9. Smoking preventive behaviors	15.06 (3.95)	0.276	0.640	0.582	0.541	0.514	0.681	0.633	0.530

*Note*. SD: Standrad deviation.

Table 4 lists the predictors of smoking preventive behaviors among participants, comparing crude and adjusted models. In Model 1 (crude), several factors were identified as significant predictors, including peer pressure (B = 1.293, *P* < 0.001) and self-image (B = 0.653, *P* < 0.001), indicating that higher levels of peer pressure and a positive self-image are associated with increased smoking prevention behaviors. Additionally, knowledge (B = 1.048, *P* < 0.001) and attitude (B = 0.540, *P* < 0.001) also played crucial roles, suggesting that greater awareness and positive attitudes toward smoking prevention significantly contribute to these behaviors. In Model 2 (adjusted), while the overall significance of peer pressure (B = 0.507, *P* < 0.001) and self-image (B = 0.235, *P* < 0.001) remained, the coefficients for knowledge (B = 0.116, *P* = 0.200) and attitude (B = 0.115, *P* < 0.001) were reduced, demonstrating that their influence may be mediated by other factors. It should be noted that belief in the consequences of smoking (B = 0.134, *P* = 0.032) and the value placed on smoking prevention (B = 0.184, *P* = 0.002) emerged as significant predictors in the adjusted model, reinforcing the importance of cognitive and value-based factors in promoting smoking preventive behaviors. The variable “friend smoker” could significantly impact smoking preventive behaviors among participants. In the crude model, the association was strong and negative (B = -3.948, 95% CI: -4.443 to -3.454, *P* < 0.001), representing that having smoker friends is linked to a substantial decrease in smoking preventive behaviors. This trend persists in the adjusted model, where the effect remains significant (B = -1.110, 95% CI: -1.560 to -0.659, *P* < 0.001). The adjusted model explains 63% of the variance in these behaviors, highlighting the multifaceted nature of influences on smoking prevention among participants.

**Table 4:** Predictors of the Smoking Preventive Behaviors Among the Participants

**Variables**	**Model 1 (Crude)**		**Model 2 (Adjusted)**	
	**B (95% CI)**	***P*** **-value**	**B (95% CI)**	***P*** **-value**
Age	-0.268 (-0.410, -0.161)	0.001	0.030 (-0.057, 0.117)	0.494
Gender	-3.961 (-4.472, -3.451)	0.001	-0.444 (-0.953, 0.065)	0.087
Academic level	-0.099 (-3.339, 0.140)	0.414	-	-
Working beside study	-2.812 (-3.473, -2.151)	0.001	-.0219 (-0.735, 0.297)	0.405
Family economic status	-0.156 (-0.781,- 0.469)	0.624	-	-
Father’s level of education	-0.083 (-0.295, 0.130)	0.445	-	-
Mother’s level of education	-0.113 (-0.375, 0.150)	0.339	-	-
Marital status	-0.568 (-1.522, 0.387)	0.243	-0.284 (-0.912, 0.344)	0.375
Residency	-0.519 (-1.-95, 0.056)	0.077	-0.208 (-0.571, 0.156)	0.262
Father smoker	-1.099 (-1.733, -0.465)	0.001	0.154 (-.0247, 0.555)	0.452
Mother smoker	-0.849 (-3.452, 1.753)	0.522	-	-
Sister smoker	-3.743 (-8.226, 0.741)	0.102	-0.040 (-2.834, 2.754)	0.977
Brother smoker	-1.189 (-1.805, -0.573)	0.001	-0.370 (-0.763, 0.023)	0.065
Friend smoker	-3.948 (-4.443, -3.454)	0.001	-1.110 (-1.560, -0.659)	0.001
Knowledge	1.048 (0.789-, 1.307)	0.001	0.116 (-0.061, 0.293)	0.200
Attitude	0.540 (0.494, 0.586)	0.001	0.115 (0.057, 0.173)	0.001
Belief	0.989 (0.890, 1.087)	0.001	0.134 (0.012, 0.257)	0.032
Value	0.918 (0.816, 1.019)	0.001	0.184 (0.068, 0.299)	0.002
Family pressure	1.278 (1.127, 1.430)	0.001	-0.025 (-0.179, -0.129)	0.751
Peer pressure	1.293 (1.194, 1.392)	0.001	0.507 (0.370, 0.644)	0.001
Self-image	0.653 (0.596, 0.710)	0.001	0.235 (0.171, 0.299)	0.001
Intention	1.204 (1.067, 1.341)	0.001	0.058 (-0.080, 0.196)	0.410

*Note*. B: Regression coefficient; CI: Confidence interval.

## Discussion

 Key findings were obtained regarding smoking behaviors among the students. Notably, 11.1% reported a history of smoking, with male students showing a significantly higher prevalence (27.9%) compared to females (1.4%). Factors influencing smoking included age, gender, and familial influences, particularly the smoking status of friends, fathers, and brothers, which were associated with increased smoking likelihood. Peer pressure and self-image emerged as significant predictors of smoking preventive behaviors.

 Our findings confirmed that 11.1% of the participants had a history of smoking, with rates significantly higher among men (27.9%) in comparison to women (1.4%). Similarly, research by Mahfouz et al on students in Saudi Arabia indicated that 8% of the students were smokers.^10^ A study conducted in Saudi Arabia revealed that 23.6% of students had smoked at some point.^25^ Similarly, a recent study in Turkey found a smoking prevalence of 29.4% among male students and 12.3% among female students, with an overall rate of 19.8%.^1^ Considering the significant health and educational impacts of smoking on students, it is essential to implement preventive interventions. To address the smoking prevalence among the students, particularly the higher rates among males, it is recommended that the authorities implement targeted smoking cessation programs and awareness campaigns in universities and integrate smoking prevention education into the curriculum. Collaboration with healthcare providers for health screenings and workshops is important, along with tailored support for female students to maintain their low smoking rates. These strategies aim to foster a healthier environment and promote smoke-free lifestyles among youth.

 Students who were in the age range of 21–23 years were found to be 2.06 times more likely to smoke compared to the 17–20-year-old group. In contrast, no significant differences were observed across other age groups. This trend may stem from the stress associated with academic pressures and the adjustment to the transition from high school to a university setting. Additionally, concerns about future work conditions might contribute to this behavior. A more comprehensive study is needed to gain deeper and more nuanced insights into these findings. The results of our study revealed that male students were 11.6 times more likely to smoke compared to female students, aligning closely with findings from similar research. Existing evidence indicates that boys tend to exhibit a greater propensity for engaging in risky behaviors. A study conducted by Kuru Sönmez et al among students in Turkey also highlighted a higher likelihood of smoking among male students.^26^ A study performed among medical students in Turkey reported that boys were 2.9 times more likely to smoke compared to girls.^27^ Similarly, research by Song et al on students in China demonstrated a higher likelihood of smoking among male students.^28^ The prevalence of tobacco use among women might be underestimated due to reporting bias, as society tends to view smoking by women as more taboo compared to men.^29^ Given the significantly higher smoking rates among male students compared to their female counterparts, it is crucial to prioritize the creation and execution of targeted smoking prevention and cessation programs for male students in Iraq. Educational planners and health policymakers should take into account the need for gender-specific approaches when designing smoking prevention initiatives for students in the country.

 The results of this study confirmed that students were 1.98 times more likely to smoke if their father smoked and 2.68 times more likely if their brother smoked. Similarly, research conducted by Legleye et al in France found that a father’s smoking raised the likelihood of students’ smoking by 1.17 times,^30^ aligning with our findings. The findings of a study performed by Martins et al among students in Timor-Leste indicated that parental smoking increased the likelihood of students smoking by 1.2 times, with the odds increased to 3.4 times if both parents smoked.^31^ Similarly, research among students in China revealed a significant connection between non-smoking students’ intentions or efforts to control tobacco use and the smoking history of their parents.^32^ A study conducted in Thailand reported that students whose parents considered smoking acceptable or who were unsure about their parents’ stance on smoking were more likely to smoke compared to those whose parents deemed smoking unacceptable.^33^ The findings of this study underline a strong correlation between parental smoking habits and the likelihood of students smoking, with students being 1.98 times more likely to smoke if their father smokes and 2.68 times more likely if their brother smokes. This emphasizes the significant impact of family attitudes and behaviors on smoking initiation among youth. To combat this issue, it is recommended that families implement strict “no smoking at home” policies to create healthier environments for students. Additionally, educational programs should be developed to promote supportive attitudes toward nonsmokers among parents and family members, highlighting the importance of their role in shaping children’s behaviors. By addressing these social environmental factors, it is possible to effectively reduce smoking prevalence among students and foster a culture of health and well-being.

 Having friends who smoke is one of the most significant factors influencing smoking behavior among college students. Based on our findings, college students with friends who smoke were 10.4 times more likely to smoke themselves. Li et al emphasized that such students are particularly susceptible to adopting smoking habits.^7^ Similarly, a study in Turkey found a strong link between having friends who smoke and an increased likelihood of smoking.^26^ In some cases, non-smoking college students began smoking due to the influence of a close friend or a family member who smoked.^34^ The role of social norms in shaping smoking behavior largely revolves around peer pressure and group integration.^35^ Adolescents often feel compelled to smoke or continue smoking when surrounded by peers who do so, driven by the need for acceptance within the group. Some researchers argue that this peer pressure serves as a tool for achieving social inclusion.^36^ The findings of this study highlight that having friends who smoke significantly increases the likelihood of smoking among college students, with those surrounded by smoking peers being 10.4 times more likely to adopt the habit themselves. This underscores the powerful influence of social norms and peer pressure on smoking behavior. To address this issue, it is recommended that authorities implement targeted smoking prevention programs that specifically focus on counteracting peer pressure and promoting positive social norms. These programs should encourage students to build supportive networks that discourage smoking and provide resources for nonsmokers to resist peer pressure. Moreover, fostering environments that celebrate non-smoking behaviors through campus initiatives and peer-led campaigns can help mitigate the influence of smoking friends, ultimately contributing to healthier choices among students.

 The results of our study confirmed that attitude, belief, and value are key predictors of smoking prevention behaviors. Similarly, a study conducted by Lin et al in China reported that social norms, positive outcome expectations, and attitudes toward tobacco control policies were strongly linked to adolescent smoking behaviors.^37^ The influence of anticipated consequences, whether positive or negative, is highlighted as a critical factor in shaping behavior. Based on the findings of our study, health policymakers and educators need to prioritize the development of programs that focus on shaping positive attitudes, beliefs, and values regarding smoking prevention. Furthermore, leveraging theoretical frameworks to evaluate attitudes and expected outcomes could provide deeper insights into behavioral change mechanisms.

 One significant factor influencing smoking prevention behaviors is peer pressure. Research highlights peer pressure as a key driver of smoking habits among college students.^38^ Studies have consistently shown that adolescent behavior is commonly shaped by peers, parental influence, and prevailing normative beliefs.^39^ Social norms surrounding smoking play a critical role in shaping tobacco control policies and research efforts. The smoking behaviors of parents and close friends have consistently emerged as significant predictors of smoking initiation among youth. Research has also indicated that disapproval of smoking can contribute to a reduction in smoking behaviors.^40^ Social norm interventions aim to provide accurate insights into peer group behaviors to address misunderstandings about such norms.^41^ As a result, the social norm surrounding smoking plays a crucial role in shaping adolescents’ smoking habits and experiences. Peer education serves as an effective strategy to influence and modify students’ smoking behaviors.^37^ Perceptions of peer-related social norms surrounding smoking have a role in influencing college students’ smoking habits. This study highlights the need for creating targeted health education programs and social norm interventions by health policymakers to address and correct misunderstandings about social norms, ultimately aiming to reduce youth smoking behaviors.

 Self-image emerged as another significant factor influencing smoking prevention behaviors. Numerous studies have established a link between self-image and participation in high-risk activities, such as substance abuse and suicide.^42-44^ For instance, research by Weiss et al revealed that students with lower self-image scores were more vulnerable to substance use.^42^ Similarly, findings by Friedman et al confirmed the correlation between negative self-image and substance use.^43^ Furthermore, Deep et al emphasized the role of positive family and peer role models in helping adolescents and adults cultivate a strong self-image, which can empower them to resist engaging in substance use.^44^ Self-image encompasses an individual’s understanding and perception of themselves, including their thoughts, beliefs, and aspirations regarding who they are and who they wish to become—their ideal self. This concept extends to areas such as physical appearance, personality traits, abilities, values, and principles, as well as how one perceives their alignment with societal expectations.^45^ This study assessed self-image in relation to smoking prevention by examining factors such as the perception of appearing unfriendly when declining a cigarette, the discourtesy of smoking in public spaces, attitudes toward smoking at social gatherings, and negative self-perceptions associated with smoking. The results indicated that a positive self-image regarding smoking prevention behaviors was a strong predictor of participation in such behaviors. These findings can inform the design of smoking prevention programs targeted at students.

 The strengths of our study lie in its relatively large sample size and the extensive range of data gathered on smoking and related factors. However, the study has limitations, including the reliance on self-reported data, which lacked biochemical validation of smoking status, and the likelihood of underreporting that may have led participants to minimize their smoking habits. Additionally, the cross-sectional design of the study prevented us from establishing causal relationships between potential determinants of smoking.

**Highlights**

- Males were 11.67 times more likely to smoke than females.
- Friends’ smoking status increased individual smoking likelihood by 10.4 times.
- Having smoking friends decreased smoking prevention efforts significantly.

## Conclusion

 Smoking habits among students were linked to factors such as age, gender, and the presence of a friend, father, or brother who smokes. These findings highlight the importance of parents recognizing the influence their smoking behaviors can have on their children’s likelihood of picking up the habit. Interventions should continue to prioritize male students as a key focus group. Furthermore, the study offers valuable insights, suggesting that interventions designed to foster negative perceptions of smoking, enhance skills to handle peer pressure, and address misconceptions about social norms could yield significant progress in preventing smoking among youth.

## Acknowledgments

 We extend our heartfelt appreciation to the students of Raparin University for their collaboration with the research team during this study. Our gratitude also goes to the Clinical Research Development Center of Motazedi Hospital in Kermanshah for their valuable consultation.

## Competing Interests

 The authors declare that they have no conflict of interests.

## Ethical Approval

 The study received approval from Kermanshah University of Medical Sciences (under the code IR.KUMS.REC.1401.388). The principles of informed consent and confidentiality were carefully observed during data collection.

## Funding

 This study was supported by Kermanshah University of Medical Sciences (project No. 4010664).
